# Supplementary material for: All-Cellulose Nanofiber-Based Sustainable Triboelectric Nanogenerators for Enhanced Energy Harvesting
Source: Polymers (Basel). 2024 Jun 24;16(13):1784. doi: 10.3390/polym16131784 (PMC11243854; doi:10.3390/polym16131784)
Supplement: Supplementary file 1 [file polymers-16-01784-s001.zip › polymers-3018830-supplementary/Supporting Information.pdf]

Supporting Information

All-Cellulose Nanofibers-Based Sustainable Triboelectric Nanogenerators for Enhanced Energy Harvesting

Mengyao Cao [1,2], Yanglei Chen [1,2], Jie Sha [1,2], Yanglei Xu [1,2], Sheng Chen [1,2,3,*] and Feng Xu [1,2,*]

1 State Key Laboratory of Efficient Production of Forest Resources, Beijing Forestry University, Beijing 100083, China
2 Beijing Key Laboratory of Lignocellulosic Chemistry, Beijing Forestry University, Beijing 100083, China; caomengyao@bjfu.edu.cn (C.M.); yanglei_chen@126.com (C.Y.); shajie123@bjfu.edu.cn (S.J.); xuyanglei@bjfu.edu.cn (X.Y.);
3 Guangxi Key Laboratory of Clean Pulp & Papermaking and Pollution Control, College of Light Industry and Food Engineering, Guangxi University, Nanning 530004, China
* Correspondence: shengchen@bjfu.edu.cn (C.S.); xfx315@bjfu.edu.cn (X.F.)

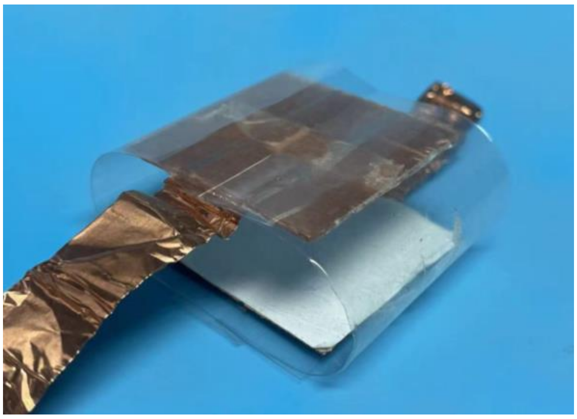

Figure S1. Photograph of the FC-TENG.

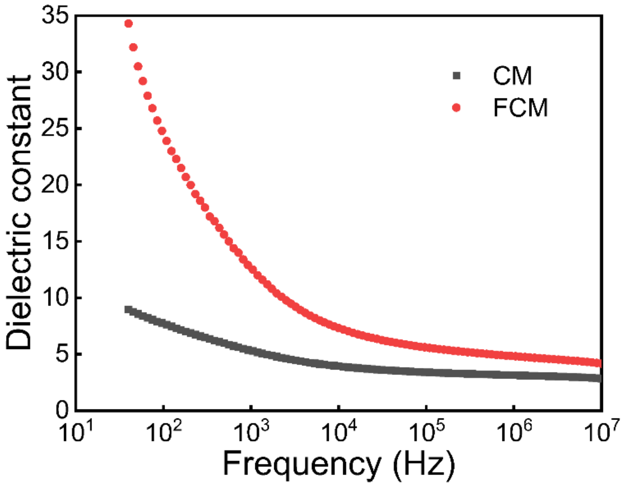

Figure S2. Dielectric constant curves of the membranes.

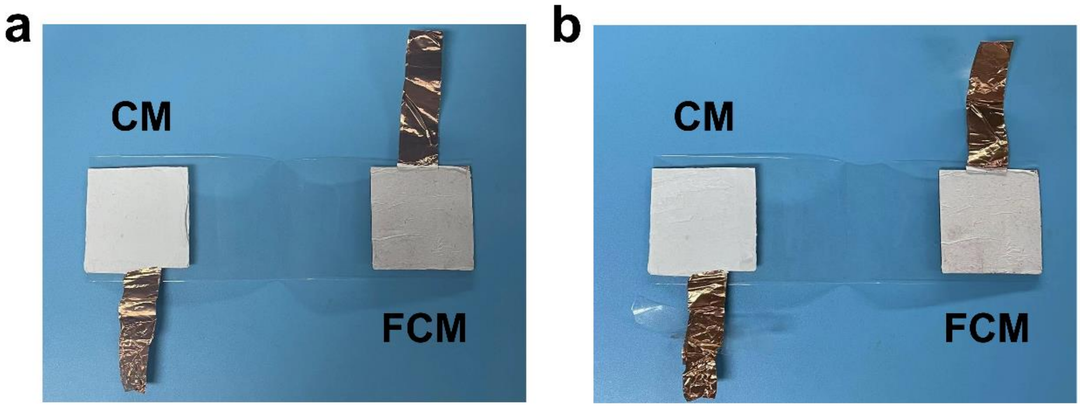

Figure S3. photographs of the FC-TENG before (a) and after (b) 15,000 recycles of operation.

Table S1. Output performance of FC-TENG with other recent reported cellulose-based TENGs.

Tribopositive	Tribonegative	Voltage (V)	Current (μA)	Power density (W/m²)	Ref.
CNF Phosphorene Hybrid Paper	Gold	5.2	/	0.018	[1]
Cellulose/PVDF/ $BaTiO_3$	PTFE	20.15	6	/	[2]
Methylated superhydrophobic CNF	FEP	120	6.1	/	[3]
PEO/PPG	PCL/EC	6.3	0.07205	2.25×10^{-6}	[4]
PEI/paper	PTFE	68.6	4.47	0.0793	[5]
polypyrrole-coated cellulose paper	Nitrocellulose	60	/	0.83	[6]
BC/$BaTiO_3$	PDMS	57.6	5.78	0.0048	[7]
Polyamide	PFOTES-CNF	28.5	9.3	0.0135	[8]
Alc-S_5-CNF	PVDF	7.9	5.13	0.182	[9]
CNF-PEI-Ag	FEP	100	1.1	0.43	[10]
**Cellulose**	**Fluorinated cellulose**	**94**	**8.5**	**0.15**	**This work**

CNF = cellulose nanofiber, PVDF = polyvinylidene fluoride, PTFE = Polytetrafluoroethylene, FEP = fluorinated ethylene propylene, PEO = polyethylene oxide, PPG = poly(propylene glycol), PCL = polycaprolactone, EC = ethyl cellulose, PEI = polyethyleneimine , BC = bacterial cellulose, PDMS = polydimethylsiloxane, PFOTES = triethoxy-1H,1H,2H,2H-tridecafluoro-n-octylsilane, Alc-S_5-CNF = allicin grafted CNFs.

References.

1. Cui, P.; Parida, K.; Lin, M.; Xiong, J.; Cai, G.; Lee, P.S. Transparent, Flexible Cellulose Nanofibril–Phosphorene Hybrid Paper as Triboelectric Nanogenerator. *Adv Materials Inter* **2017**, *4*, 1700651, doi:10.1002/admi.201700651.

2. Sun, Z.; Yang, L.; Liu, S.; Zhao, J.; Hu, Z.; Song, W. A Green Triboelectric Nano-Generator Composite of Degradable Cellulose, Piezoelectric Polymers of PVDF/PA6, and Nanoparticles of BaTiO3. *Sensors* **2020**, *20*, 506, doi:10.3390/s20020506.
3. Zhang, C.; Zhang, W.; Du, G.; Fu, Q.; Mo, J.; Nie, S. Superhydrophobic Cellulosic Triboelectric Materials for Distributed Energy Harvesting. *Chemical Engineering Journal* **2023**, *452*, 139259, doi:10.1016/j.cej.2022.139259.
4. Li, C.; Luo, R.; Bai, Y.; Shao, J.; Ji, J.; Wang, E.; Li, Z.; Meng, H.; Li, Z. Molecular Doped Biodegradable Triboelectric Nanogenerator with Optimal Output Performance. *Adv Funct Materials* **2024**, 2400277, doi:10.1002/adfm.202400277.
5. Wu, S.; Li, G.; Liu, W.; Yu, D.; Li, G.; Liu, X.; Song, Z.; Wang, H.; Liu, H. Fabrication of Polyethyleneimine-Paper Composites with Improved Tribopositivity for Triboelectric Nanogenerators. *Nano Energy* **2022**, *93*, 106859, doi:10.1016/j.nanoen.2021.106859.
6. Shi, X.; Chen, S.; Zhang, H.; Jiang, J.; Ma, Z.; Gong, S. Portable Self-Charging Power System via Integration of a Flexible Paper-Based Triboelectric Nanogenerator and Supercapacitor. *ACS Sustainable Chem. Eng.* **2019**, *7*, 18657–18666, doi:10.1021/acssuschemeng.9b05129.
7. Jakmuangpak, S.; Prada, T.; Mongkolthanaruk, W.; Harnchana, V.; Pinitsoontorn, S. Engineering Bacterial Cellulose Films by Nanocomposite Approach and Surface Modification for Biocompatible Triboelectric Nanogenerator. *ACS Appl. Electron. Mater.* **2020**, *2*, 2498–2506, doi:10.1021/acsaelm.0c00421.
8. Nie, S.; Fu, Q.; Lin, X.; Zhang, C.; Lu, Y.; Wang, S. Enhanced Performance of a Cellulose Nanofibrils-Based Triboelectric Nanogenerator by Tuning the Surface Polarizability and Hydrophobicity. *Chemical Engineering Journal* **2021**, *404*, 126512, doi:10.1016/j.cej.2020.126512.
9. Roy, S.; Ko, H.-U.; Maji, P.K.; Van Hai, L.; Kim, J. Large Amplification of Triboelectric Property by Allicin to Develop High Performance Cellulosic Triboelectric Nanogenerator. *Chemical Engineering Journal* **2020**, *385*, 123723, doi:10.1016/j.cej.2019.123723.
10. Zhang, C.; Lin, X.; Zhang, N.; Lu, Y.; Wu, Z.; Liu, G.; Nie, S. Chemically Functionalized Cellulose Nanofibrils-Based Gear-like Triboelectric Nanogenerator for Energy Harvesting and Sensing. *Nano Energy* **2019**, *66*, 104126, doi:10.1016/j.nanoen.2019.104126.
